# Influence of Technological Factors on the Interlayer Bond Strength of 3D-Printed Concrete

**DOI:** 10.3390/ma19173641

**Published:** 2026-08-27

**Authors:** Leonid Dvorkin, Vitaliy Marchuk, Ruslan Makarenko, Yuri Ribakov

**Affiliations:** 1 Institute of Civil Engineering and Architecture, National University of Water and Environmental Engineering, 33028 Rivne, Ukrainev.v.marchuk@nuwm.edu.ua (V.M.);; 2 Department of Civil Engineering, Ariel University, Ariel 40700, Israel

**Keywords:** interlayer bond strength, adhesive bond strength, splitting tensile strength, water demand, extrusion, mathematical models

## Abstract

This paper presents an analysis of mathematical models developed from experimental data to describe the effects of concrete mixture workability, the time interval between the deposition of adjacent layers, aggregate size, and concrete mixture composition on the interlayer bond strength of 3D-printed concrete. The models enable a quantitative assessment of the nature and degree of influence of the investigated technological factors on the interlayer bond strength of concrete. The results demonstrate that the interlayer bond strength, evaluated based on concrete splitting tensile strength, exhibits an extremal relationship. The range of optimal technological parameters within which the maximum interlayer bond strength is achieved has been determined. The effects of interactions between the investigated technological factors on the interlayer bond strength are also examined. The influence of these factors on interlayer bond strength is associated with their effect on the concrete mixture water demand, which is described by the developed mathematical model. In addition, a relationship between the interlayer bond strength of concrete and the water-to-cement ratio is established.

## 1. Introduction

In 3D concrete printing technology, one of the key indicators of the quality of printed structures is interlayer bond strength, as it ensures the structural integrity and monolithic behavior of the printed elements. Unlike conventional concrete casting, in which the material structure is formed simultaneously throughout the entire volume, additive manufacturing builds the structure layer by layer, resulting in the formation of potentially weak interfaces between adjacent layers. Insufficient adhesion at these interfaces may lead to too high anisotropy of the mechanical properties, as well as reduced load-bearing capacity and durability of structural elements [1].

The interlayer bond strength can be appropriately evaluated using the splitting tensile strength, which quantitatively characterizes the ultimate bond strength at the interface between adjacent layers. This approach enables an objective assessment of the adhesive performance of the concrete mixture, effectiveness of modifying admixtures, and influence of printing parameters on the formation of structural bonds at the interfaces between adjacent layers.

The quality of the interlayer region, which governs the strength and durability of extruded structures, is influenced by a wide range of factors [2]. The most significant of these include the time interval between the successive layers’ deposition, the rheological properties of the concrete mixture, ambient temperature and humidity, printing speed, and the deposited layers’ height and width.

The concrete mixture’s rheological properties can change significantly with variations in the maximum aggregate particle size [3]. Studies have shown that the use of aggregates with an excessively large maximum particle size may lead to defects such as segregation and the formation of localized voids [4]. This factor is particularly critical in thin-layer extrusion, where coarse particles may hinder uniform material compaction and promote the formation of microcracks. Reducing the maximum aggregate particle size improves the homogeneity of the extruded layer and enhances its surface quality. Fine aggregates provide better particle packing and reduce the risk of segregation. However, an excessive reduction in particle size increases the mixture water demand, resulting in higher binder consumption and adversely affecting the strength and shrinkage behavior of the material.

**Figure 1:**
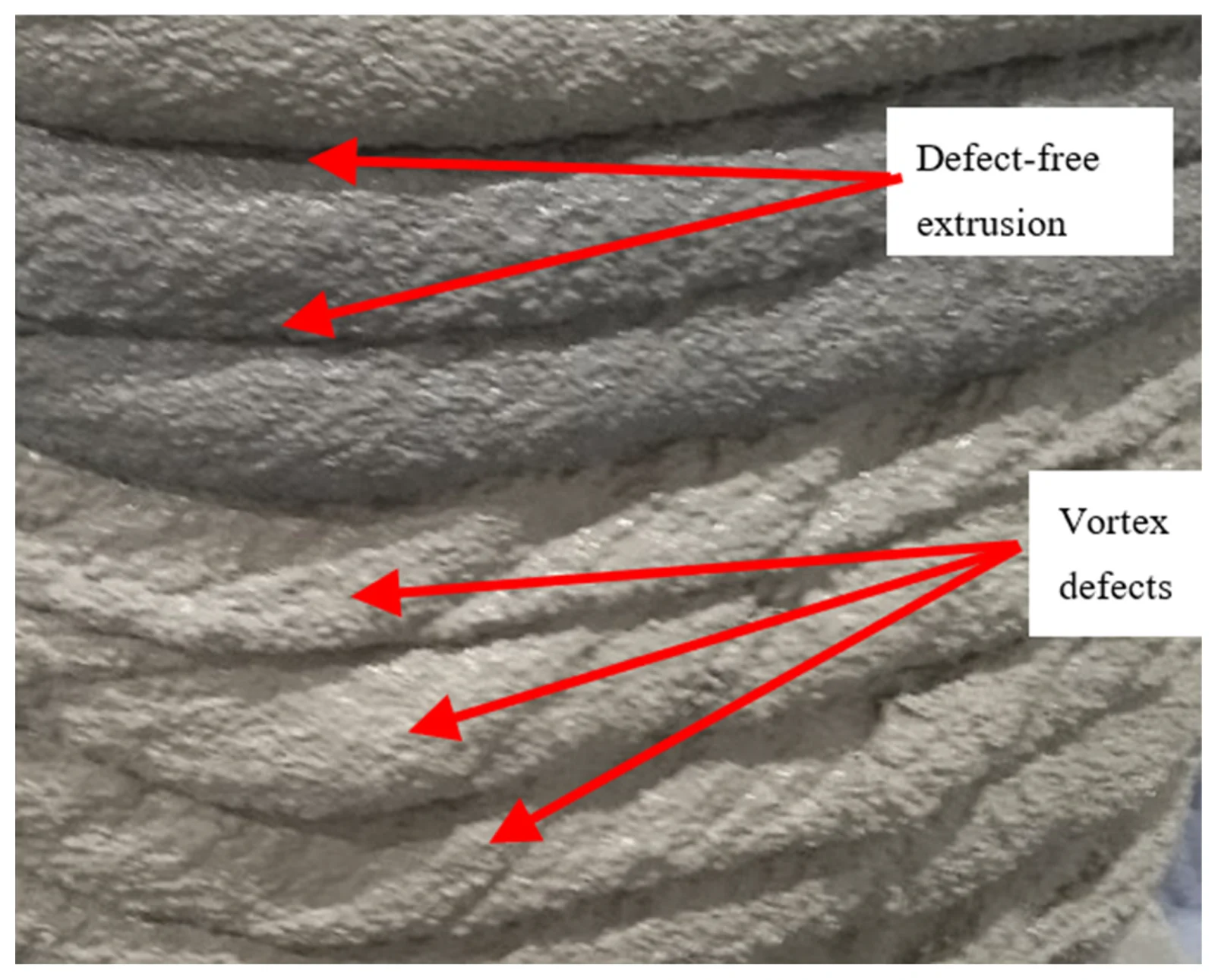
Defects formed during layer deposition as a function of aggregate particle size.

The aggregate particle size is directly related to the concrete mixture properties, particularly its plasticity and workability. As the aggregate particle size changes, the mixture water demand must be adjusted to maintain adequate extrudability [5,6]. In addition, the cement paste content plays a crucial role, acting as a lubricant that reduces friction against the nozzle inner surface and promotes a more uniform mixture flow through the nozzle cross-section, thereby minimizing extrusion defects (Figure 1) [7].

Despite the considerable number of studies investigating the influence of technological factors on the quality of the interlayer region in 3D-printed concrete, most do not provide a quantitative assessment of the effects of individual factors or their interactions. This limitation complicates the prediction of the combined influence of technological parameters on the quality of the interlayer region of extruded concrete and, consequently, on the performance of products manufactured under specific 3D printing conditions. Therefore, the objective of this study is to develop and analyze quantitative relationships, expressed as mathematical models, describing the influence of the principal technological factors on the interlayer bond strength of extruded concrete. The results of this investigation are presented in this paper.

## 2. Materials and Methods

Fine-grained concrete was used in this study. The constituent materials included Portland cement CEM I 52.5N from Cement Plant “VIPCEM” by CRH, Zdolbuniv, Ukraine, and quartz sand with a maximum particle size of 2.5 mm, a fineness modulus M_F_ = 2.04, and a content of dusty and clay impurities of 1.8%. The chemical and mineralogical composition and the Portland cement strength are presented in Table 1 and Table 2.

To investigate the effect of the maximum aggregate particle size on the interlayer bond strength, quartz sand with three particle size ranges was used: 0.16–0.5 mm, 0.16–1.5 mm, and 0.16–2.5 mm. The required fractions were obtained by sieving the original sand through the corresponding standard sieves.

**Figure 2:**
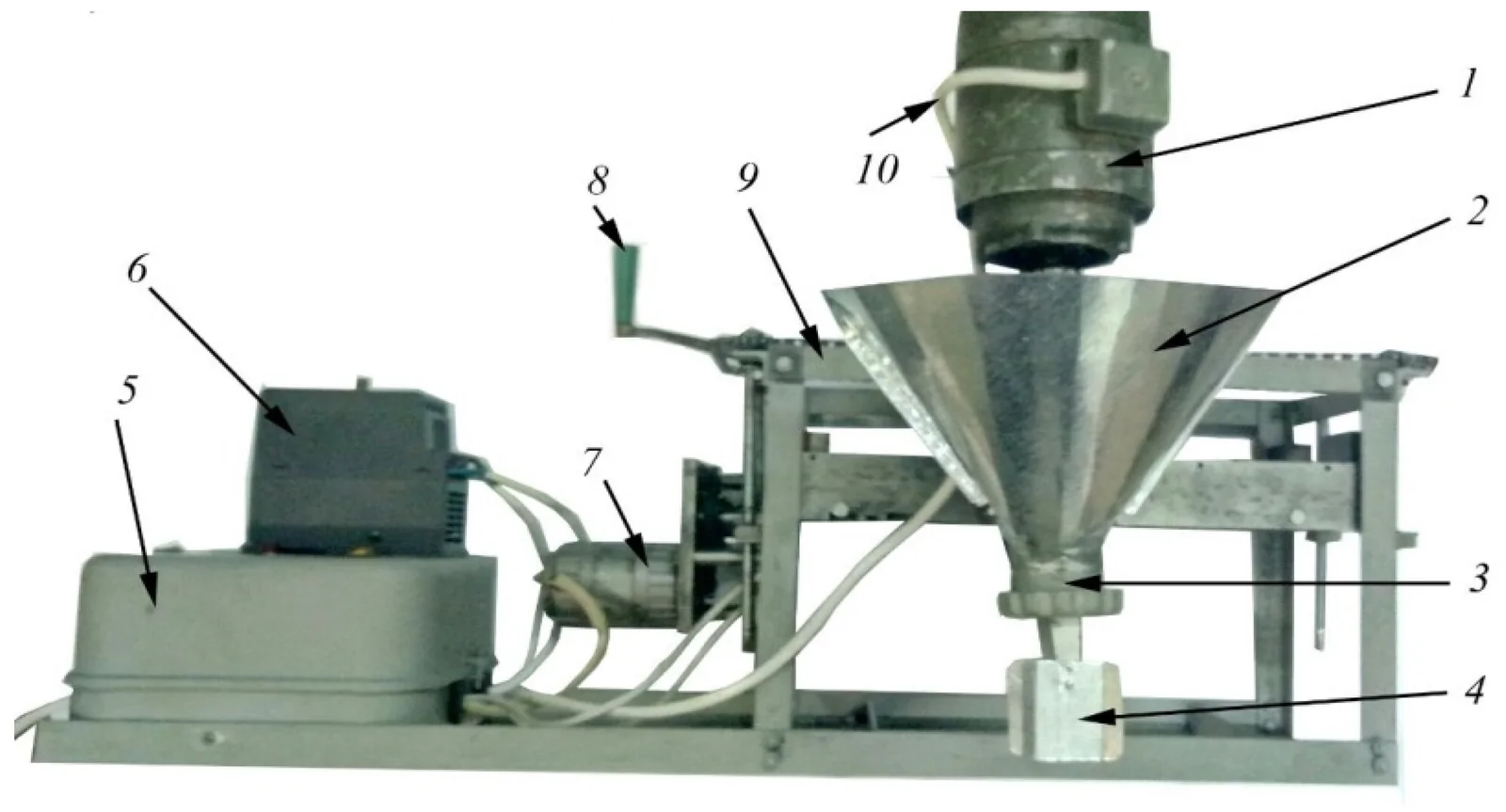
Laboratory 3D printer: 1—extruder drive motor; 2—concrete mixture hopper; 3—screw auger; 4—interchangeable nozzle; 5—control panel; 6—variable-frequency drive (VFD); 7—reversible motor for horizontal movement of the extruder; 8—manual drive for vertical movement of the extruder; 9—frame; 10—power cable for the drive motors.

The specimens were produced using a laboratory 3D printer (Figure 2). The concrete mixtures had a workability, determined by the flow table test, in the range of 200–240 mm. The layer width was 45 mm, and the height was 15 mm, which corresponds to the dimensions of the 3D printer. A polycarboxylate-based superplasticizer, Melflux 2651F, BASF Construction Solutions GmbH, Trostberg, Germany, with a water-reducing effect of 30%, was incorporated into concrete mixtures at a dosage of 0.2% by mass of cement.

**Figure 3:**
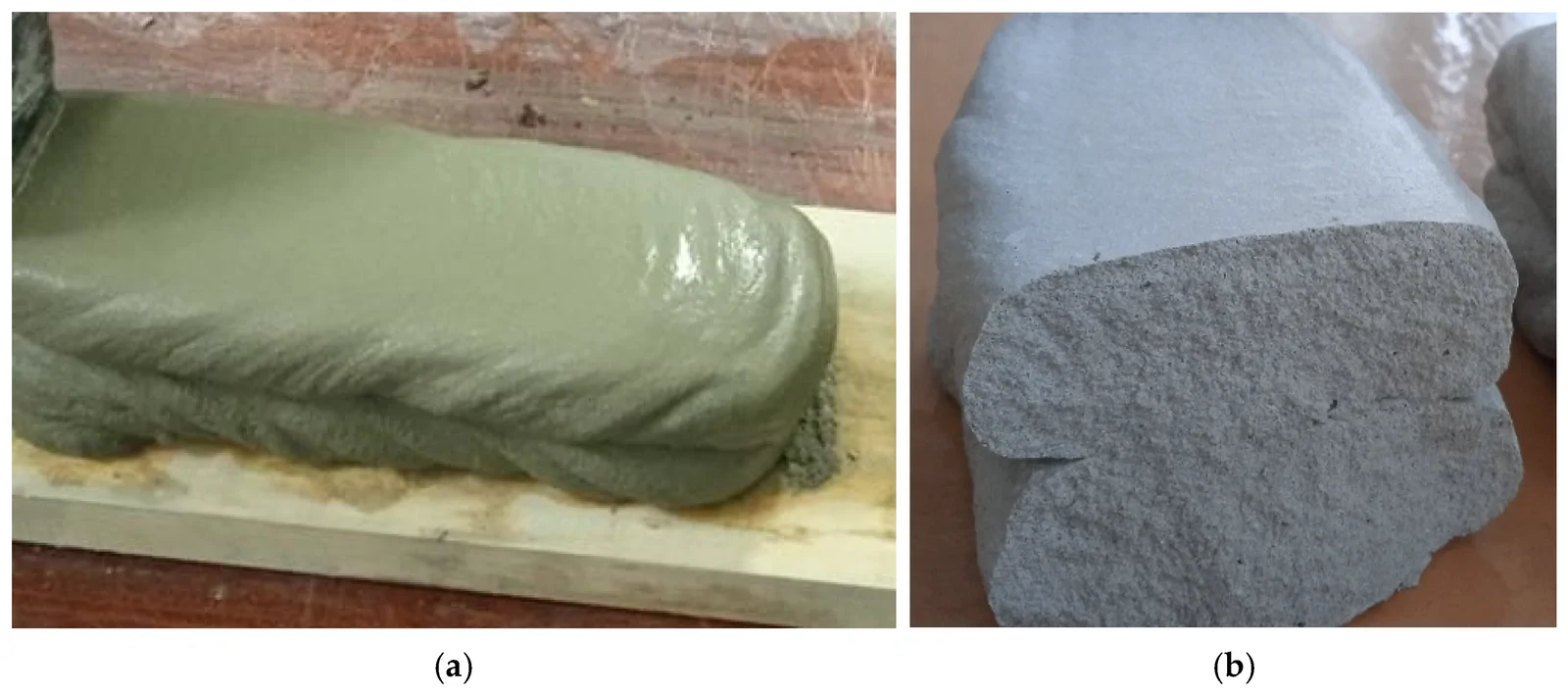
Specimens used for determining the splitting tensile strength: (**a**) after extrusion (immediately after production); (**b**) before testing.

The extruded two-layer specimens (Figure 3a) were cured under standard conditions for 24 ± 2 h at a temperature of 18–22 °C, protected from moisture evaporation, and then placed in a chamber with a relative humidity of >95% and a temperature of 20 ± 3 °C for 27 days prior to testing. Three 60 mm-long samples were then cut from the printed elements and tested for splitting tensile strength, as shown in Figure 3b.

The nozzle travel speed was maintained constant and typical for industrial printers—150 mm/s.

The interlayer bond strength of the extruded concrete was evaluated in terms of the splitting tensile strength at the boundary of layers at the layer boundary under load application perpendicular to the printing direction.

The equation used to calculate the splitting tensile strength considering a rectangular cross-section was:

(1) $$f_{ctn}=\frac{2\cdot F}{\pi \cdot L\cdot H}$$

where $f_{ctn}$—is the splitting tensile strength, in megapascals (MPa or N/mm^2^).

*F*—is the maximum breaking force (load) applied to the specimen, in newtons (N).

*L*—is the length of the contact line in millimeters (mm).

*H*—is the specified transverse dimension of the specimen in millimeters (mm).

*π*—is the mathematical constant (approximately 3.14159). The study employed a mathematical experiment planning methodology [8]. Design B_2_ is a two-factor, three-level design that yields a quadratic polynomial model. Based on this approach and under the specified experimental conditions, polynomial mathematical models describing the interlayer bond strength of concrete were developed.

## 3. Experimental Results and Discussion

### 3.1. Effect of Concrete Mixture Workability and Time Interval Between the Laying of Successive Layers

The 3D-printed concrete structure formation takes place under conditions of constrained rheological properties of the mixture, limited contact time between adjacent layers, and in the absence of additional compaction, making the material properties highly sensitive to the printing process parameters. Among these parameters, concrete mixture workability and the time interval between the laying of successive layers are of particular importance [9,10,11,12,13]. Workability governs the material rheological behavior during extrusion, the ability of the mixture to establish continuous contact between adjacent layers, and the retention of the designed geometry after laying [14]. The time interval between the deposition of successive layers directly affects the surface condition of the underlying layer at the time of contact, the degree of structural strength development, and the formation of interlayer bonds [5,15].

An increase in workability improves wetting and contact between adjacent layers; however, it also increases the printed layer tendency to spread and lose its shape stability [16]. Conversely, increasing the time interval between layering promotes the development of structural strength in the underlying layer but reduces its ability to form a strong interlayer bond with the subsequent layer [17]. It was demonstrated that the interlayer bond strength is directly dependent on both the time interval between layering and the printing speed [18]. Therefore, these factors have opposing effects, creating a technological trade-off between the rheological suitability of the concrete mixture and the structural integrity of the printed element.

The time interval between the successive layers’ printing plays a crucial role in achieving the required physical and mechanical properties of the printed structural element [1]. A prolonged delay, particularly when rapid-hardening mixtures are used, may result in the formation of numerous voids, significantly reducing the printed component’s structural integrity. Such voids occur more frequently in structures produced with extended time intervals between successive layers. This phenomenon is primarily associated with the loss of surface moisture from the previously deposited layer, leading to the formation of a cold joint, which reduces the interlayer bond strength. Consequently, systematic experimental investigations aimed at quantitatively evaluating the influence of concrete mixture workability and the time interval between layers on the quality characteristics of structures produced by 3D concrete printing are of considerable practical importance.

The experimental conditions and the design matrix are presented in Table 3 and Table 4, respectively. The mixture compositions and corresponding experimental results are presented in Table 5. The water-cement ratio was W/C = 0.5.

After statistical analysis of the experimental data, the following mathematical model describing the splitting tensile strength of concrete (at 28 days (*f_c,tn_*, MPa)) was obtained:

*f_c__,__tn_* = 2.37 + 0.04X_1_ − 0.75X_2_ − 0.25X_1_^2^ − 0.64X_2_^2^ − 0.001X_1_X_2_ (2)

Analysis of the developed model indicates that the time interval between layers (X_2_) is the dominant factor adversely affecting the splitting tensile strength. As the time interval between the successive layers extrusion increases, the underlying layer undergoes partial setting, resulting in a reduced capacity for diffusion bonding and formation of a weakened interlayer interface.

The quadratic term for flowability (X_1_^2^ = −0.25) indicates that its effect is extremal. There is an optimum range of mixture workability at which the splitting tensile strength reaches its maximum, whereas deviations toward either insufficient or excessive workability result in a reduction in strength. The quadratic term associated with the time interval between layering (X_2_^2^ = −0.64) demonstrates a pronounced decrease in bond strength as the time interval deviates from its optimum value. This behavior is attributed to the rapid development of structural strength in the underlying layer and the surface film formation.

It is worth noting that destruction occurs at low strength values (up to 2 MPa) only through the interlayer surface, and at higher strength values (more than 2 MPa) partial destruction beyond it is also possible.

**Figure 4:**
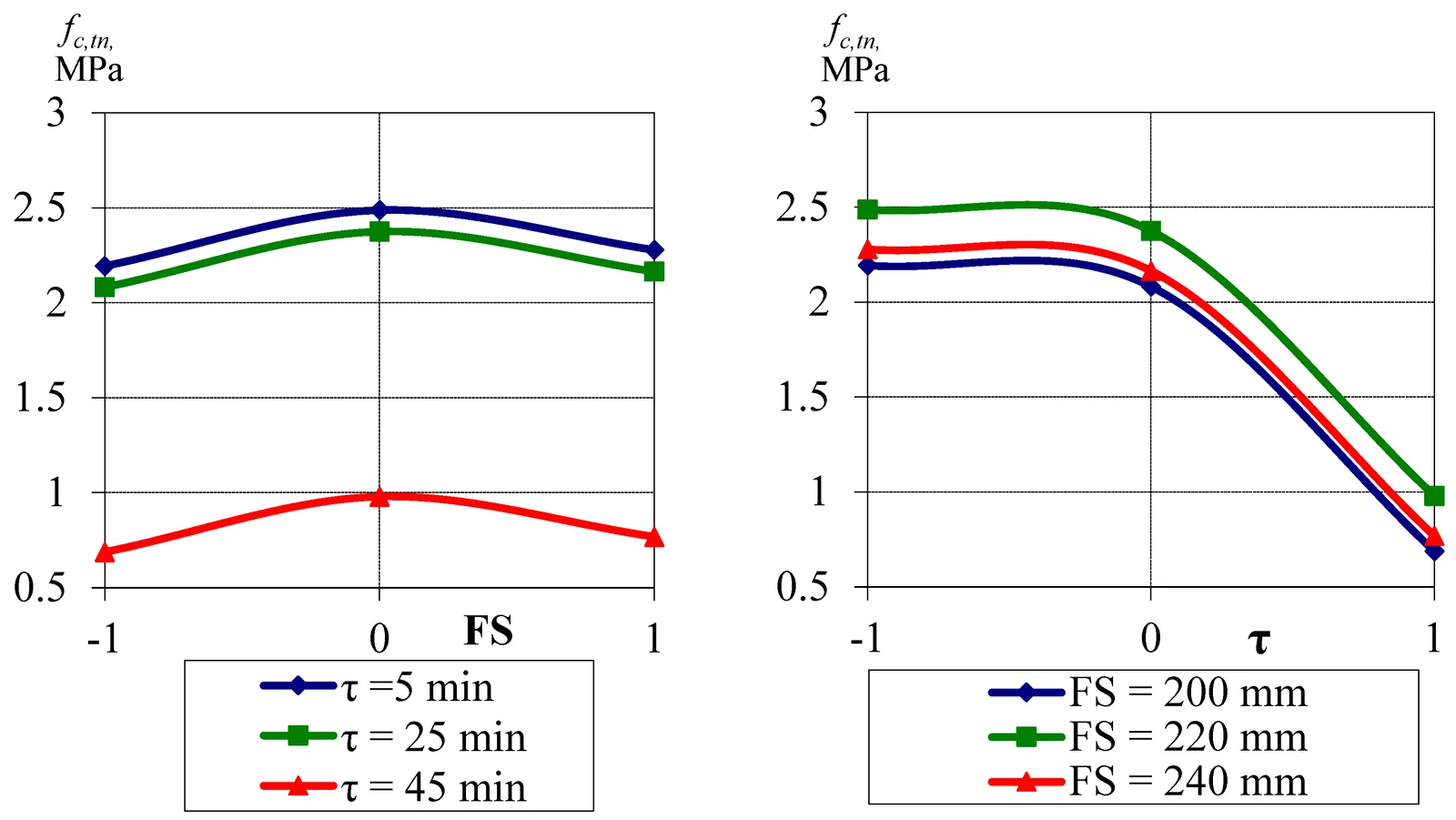
The influence of technological parameters on the splitting tensile strength of concrete (*f_c.tn_*).

Analysis of the splitting tensile strength data for concrete (Table 5) indicates that the acceptable range of mixture workability is relatively wide, whereas even a delay of several minutes in the interval between layer depositions results in a significant reduction in strength. The zone of maximum strength values is localized within the flowability ranges (FS) ≈ 215–225 mm and τ ≈ 5–10 min, which corresponds to a technologically rational extrusion regime (Figure 4). A slight reduction in the optimal time between applied layers compared to the mathematical optimum (~13 min) is necessary to ensure the quality of the extruded layers.

**Figure 5:**
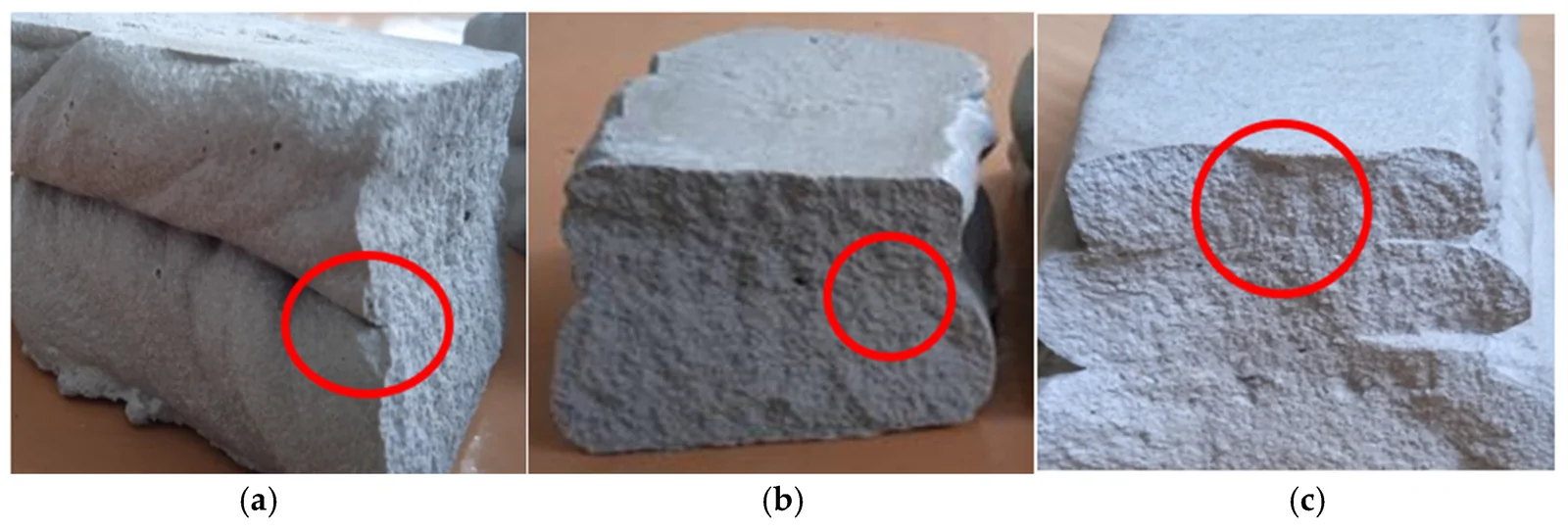
Interlayer space filling during layering: (**a**) FS = 200 mm, (**b**) FS = 220 mm, (**c**) FS = 240 mm.

At low workability values (Figure 5a), the extruded layer retains its specified geometric shape, is characterized by clearly defined boundaries, and has a limited spreading width. With increasing workability, the contact area between the deposited layer and the underlying surface increases, accompanied by a reduction in layer height and higher lateral spreading (Figure 5b). This effect results from the predominance of viscoplastic flow over the elastic–plastic behavior of the material during the initial period following extrusion.

Excessive workability leads to a loss of layer shape stability, manifested by layer flattening, smoothing of lateral surfaces, and reduced definition of the boundaries between adjacent layers. As a result, the actual geometry of the printed element deviates from the designed one, which may negatively affect printing accuracy and the load-bearing capacity of the structure.

Thus, the spreading of the extruded layer with increasing workability has a dual effect: on one hand, it promotes improved contact between layers and enhances interlayer adhesion; on the other hand, it reduces the material’s shape-retention capability. This confirms the necessity of selecting an optimum concrete mixture workability that provides a balance between rheological ability to form interlayer bonding and the geometric stability of the printed layers.

The effect of the time interval between layer depositions (cross-section at X_1_ = 0) exhibits a pronounced decreasing trend, confirming the dominant role of this factor.

**Figure 6:**
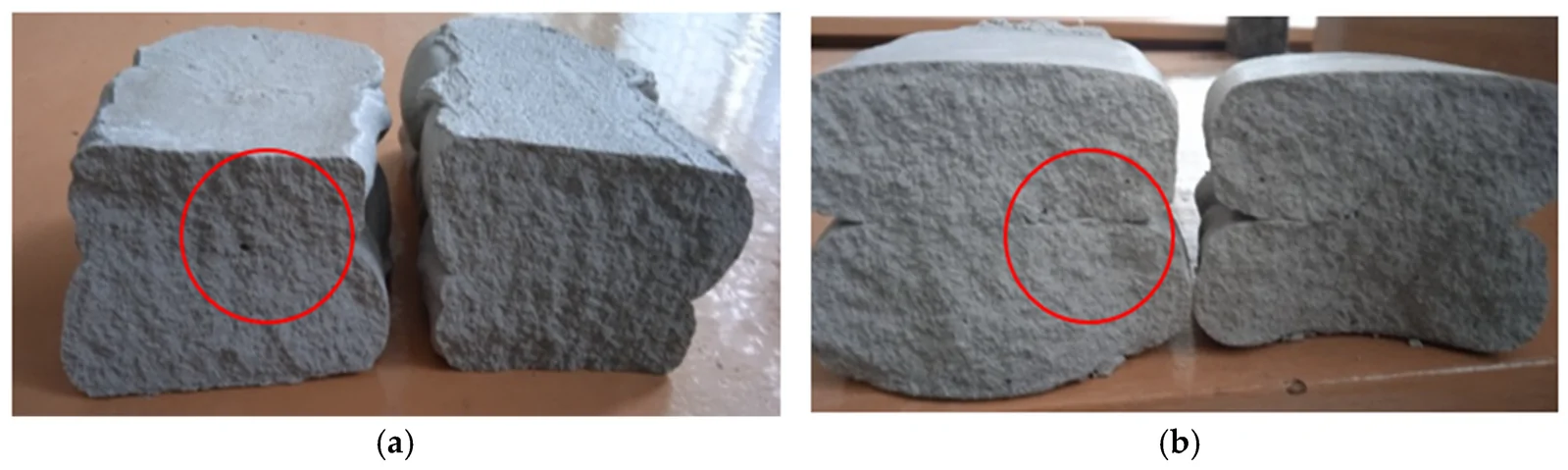
Illustration of interlayer space filling during layer deposition: (**a**) after 5 min, (**b**) after 45 min.

The minimum interlayer time during extrusions (τ ≈ 5 min) ensures the formation of interlayer bonds through plastic deformation of the underlying layer and partial diffusion of the cement paste (Figure 6a). As the time increases to 25 min, the conditions approach the state of optimal interlayer contact. A further increase in τ up to 45 min is accompanied by intensive development of the underlying layer structural strength and formation of a weakened contact interface, resulting in a reduction of the contact area and a significant decrease in the splitting tensile strength (Figure 6b).

The absence of significant interaction effects between the factors confirms the independence of their influence within the investigated range and allows the justification of optimal technological parameters for 3D concrete printing.

### 3.2. Effect of Maximum Aggregate Particle Size and the Portland Cement–Sand Ratio in the Mixture

The determining factor in the composition of fine-grained concrete mixtures for 3D printing is the maximum size of the aggregate grains and the ratio of Portland cement and aggregate consumption. The experimental design conditions are presented in Table 6.

The investigated concrete mixtures were suitable for extrusion-based fabrication using the laboratory 3D printer without formation of cracks, layering defects, or other imperfections along the length of the printed element [17,19,20,21,22,23]. The mixtures maintained a constant flowability, determined by the flow table test, within the range of 210–220 mm, while the required water demand was adjusted accordingly. The mixture feeding rate and nozzle movement speed were selected as 150 mm/s.

The experimental design matrix is presented in Table 7, while the mixture compositions and quantitative evaluation of the experimental results are given in Table 8.

The mathematical models describing the influence of the varied factors are:

Mixture water demand (W, *l*)

W = 145.93 − 10.27X_1_ + 8.4X_2_ + 0.24X_1_^2^ − 3.76X_2_^2^ − 1.65X_1_X_2_ (3)

Splitting tensile strength (*f_c,tn_*, MPa)

*f_c__,__tn_* = 2.61 − 0.18X_1_ + 0.26X_2_ − 0.32X_1_^2^ − 0.13X_2_^2^ − 0.05X_1_X_2_ (4)

The obtained model indicates that the minimum water demand of the mixture suitable for extrusion-based fabrication is achieved at a moderately increased maximum aggregate particle size of approximately 1.5–2.0 mm and a rational Portland cement content close to the average value of the variation range, approximately 0.38–0.43.

Such parameters ensure extrusion stability, reduce the risk of segregation, improve layer geometric accuracy, increase shape stability (structural strength) [14,17,24], and enhance interlayer adhesion.

Analysis of the mathematical model for splitting tensile strength (4) shows that the influence of the maximum aggregate particle size is characterized by negative linear (X_1_ = −0.13) and quadratic (X_1_^2^ = −0.06) coefficients. These values indicate that an increase in the maximum aggregate size leads to a decrease in splitting tensile strength, and increasing Dmax results in a certain reduction in adhesion. This effect can be explained by the increased heterogeneity of the contact zone, as larger aggregate particles hinder dense interlayer contact and reduce the effective area of cement paste bonding. However, in this case, an increase in particle size may also contribute to improved interlayer adhesion due to the formation of a rougher surface.

The influence of the Portland cement content, represented by the factor X_2_, indicates that an increase in its parameter within the investigated variation range promotes higher interlayer splitting tensile strength. This is attributed to an increase in the amount of cement paste in the contact zone and improved wetting of the previously laid surface.

Unlike the effect on water demand, within the investigated range, no negative influence of excessive Portland cement content on adhesion was observed.

The negative interaction coefficient (−0.04) between factors X_1_ and X_2_ indicates that the positive effect of increasing cement content decreases with increasing maximum sand particle size. Thus, at Dmax = 0.5–1.0 mm, increasing X_2_ effectively improves adhesion, whereas at Dmax = 1.5–2.5 mm, the effect of introducing a higher binder content (≥0.45) is partially reduced due to the formation of structural defects in the contact zone. This means that maximum adhesion is achieved only through a balanced combination of factors rather than by reaching extreme values of each factor individually.

Therefore, the adhesion between laid layers during 3D printing changes significantly and nonlinearly, as it is directly related to the condition of the “previous layer–subsequent layer” contact zone, water demand, and rheological characteristics of the mixture.

For mixtures suitable for 3D printing, optimum adhesion is achieved at Dmax ≤ 1.5 mm, although this may be accompanied by increased water demand, which should be compensated for by the introduction of superplasticizing additives.

Comparison and correlation of the obtained models demonstrate the need for a technological compromise because:

- minimum water demand is achieved at higher Dmax = 1.5–2.5 mm and moderate Portland cement consumption (CEM–sand ratio = 0.33–0.40)
- maximum splitting tensile strength is achieved at lower Dmax = 0.5–1.5 mm and increased Portland cement content.

This confirms that it is impossible to simultaneously optimize both investigated technological parameters at a single point within the factor space.

Considering models (3) and (4), the rational region is defined as Dmax ≈ 1.0–1.5 mm and a Portland cement–sand ratio ≈ 0.40–0.45, within which acceptable water demand, extrusion stability, and increased splitting tensile strength are ensured.

**Figure 7:**
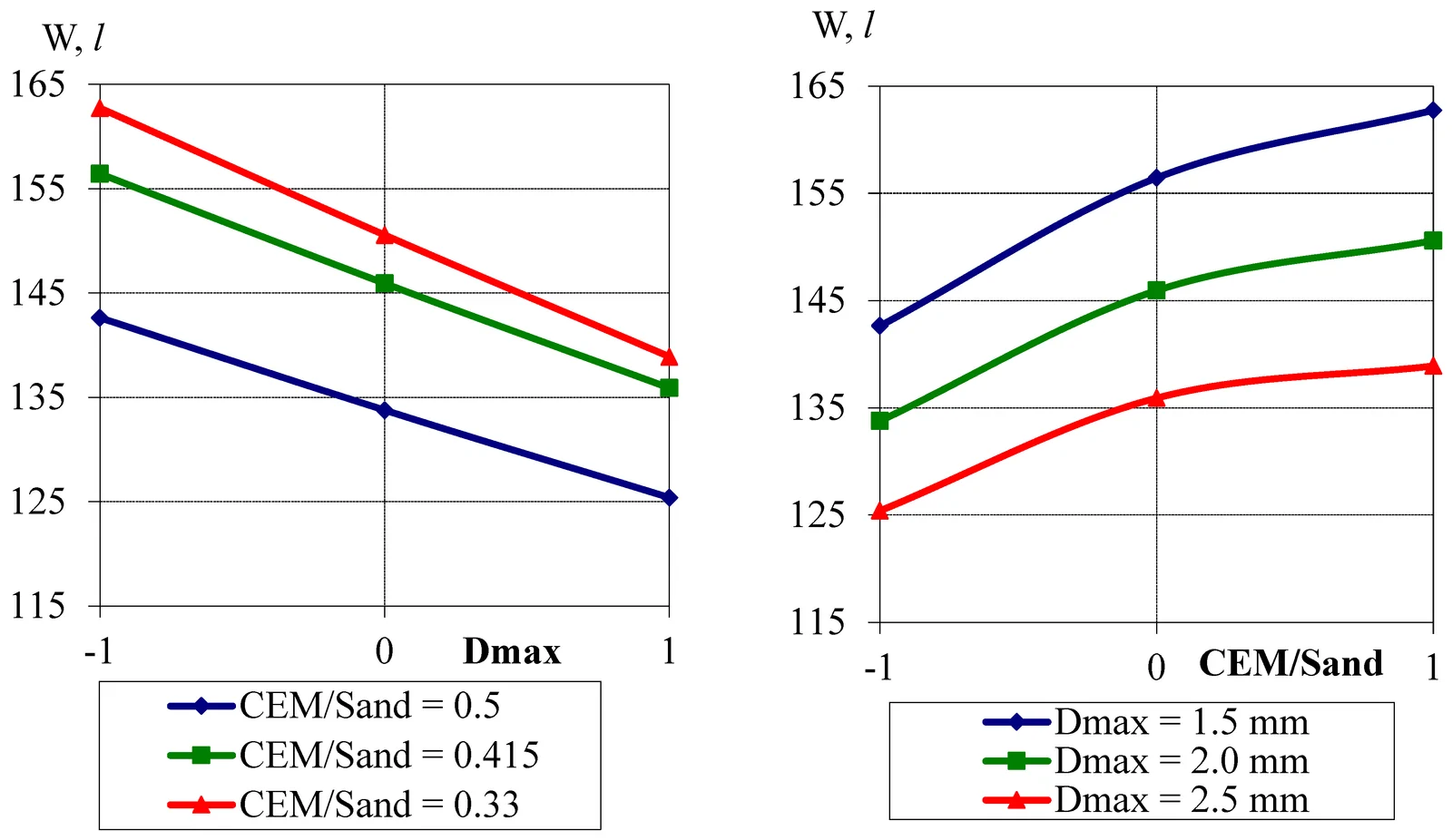
The influence of technological parameters on the concrete mixture water demand.

**Figure 8:**
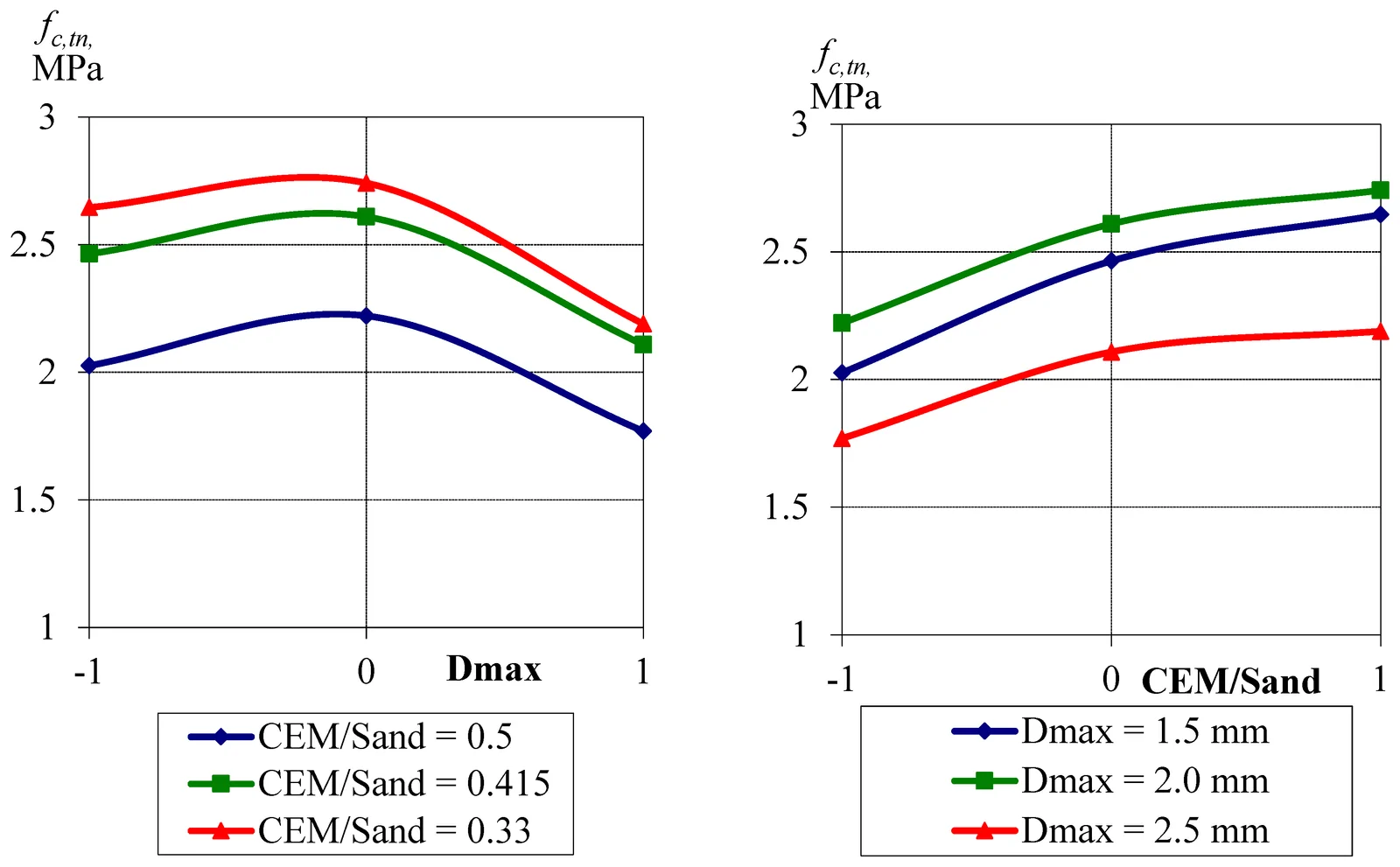
The influence of technological parameters on the splitting tensile strength of concrete.

For a more comprehensive representation of the obtained experimental data, two-factor graphs were constructed to illustrate the influence of technological factors on the mixture water demand (Figure 7) and splitting tensile strength (Figure 8).

## 4. Conclusions

Analysis of the obtained mathematical models for the splitting tensile strength of fine-grained 3D-printed concrete enables comparative quantitative assessment of the effects of setting tensile strength, mixture workability, time interval between layer deposition, maximum aggregate size, and the aggregate-to-Portland cement ratio on this parameter.

Within the studied ranges of the considered technological factors, as a result of changing the corresponding conditions for the structure formation of concrete extruded using additive technology, it is possible to determine the region of optimal values that allow obtaining the maximum splitting tensile strength.

Considering the obtained models, a compromise region of concrete mixtures’ water demand values and corresponding mixture compositions, ensuring a sufficiently high splitting tensile strength of extruded concrete, was determined. Thus, the proposed methodology enables the effective design of concrete compositions for 3D-printed elements, thereby reducing the consumption of natural resources and labor costs in construction.

## Figures and Tables

**Table 1:** Chemical–mineralogical composition of Portland cement clinker *.

Mineral Content, %				Oxide Content, %							
C_3_S	C_2_S	C_4_AF	C_3_A	SiO_2_	Al_2_O_3_	Fe_2_O_3_	CaO	MgO	SO_3_	K_2_O	Na_2_O
57.1	21.27	12.19	6.87	21.80	5.32	4.11	66.80	0.95	0.63	0.54	0.42

* The chemical composition of Portland cement based on the used clinker was distinguished by an additional SO_3_ content due to the introduction of gypsum in the amount of 3.1%.

**Table 2:** Physical and mechanical characteristics of cement CEM I 52.5N.

No.	Property	Unit	Values
1	Density (specific gravity)	kg/m^3^	3100–3150
2	Bulk density	kg/m^3^	1050–1150
3	Blaine specific surface area	m^2^/kg	430–450
4	Normal consistency	%	28–29.5
5	Initial setting time	min	120–135
6	Final setting time	min	165–185
7	Compressive strength at 2 days	MPa	29.3–31.4
8	Compressive strength at 28 days	MPa	53.5–55.2

**Table 3:** Experimental design conditions for workability tests.

Technological Factors		Variation Levels			Variation Interval
Natural Value	Coded Form	−1	0	+1	
Mixture flowability (flow spread), mm (FS)	X_1_	200	220	240	20
Time interval between layers, min (τ)	X_2_	5	25	45	20

**Table 4:** Experimental design matrix for workability tests.

No.	Coded Values		Natural Values	
	X_1_	X_2_	FS, mm	τ, min
1	1	1	240	45
2	1	−1	240	5
3	−1	1	200	45
4	−1	−1	200	5
5	1	0	240	25
6	−1	0	200	25
7	0	1	220	45
8	0	−1	220	5
9	0	0	220	25
10	0	0	220	25
11	0	0	220	25

**Table 5:** Mixture compositions and experimental results for workability tests.

No.	Mixture Composition, kg/m^3^				Splitting Tensile Strength at 28 Days, MPa	Standard Deviation, s	Standard Error, $s_{\bar{x}}$	Coefficient of Variation, V, %
	CEM *	Sand	SP **	Water				
1	600	1339	1.2	300	0.82	0.07	0.040	8.88
2	550	1448	1.1	275	2.24	0.11	0.061	4.81
3	600	1339	1.2	300	0.75	0.06	0.036	8.92
4	550	1448	1.1	275	2.17	0.15	0.087	7.38
5	600	1339	1.2	300	2.15	0.13	0.074	6.02
6	550	1448	1.1	275	2.05	0.10	0.060	4.96
7	570	1404	1.14	285	0.87	0.06	0.033	6.28
8	570	1404	1.14	285	2.55	0.15	0.087	5.67
9	570	1404	1.14	285	2.40	0.20	0.117	7.87
10	570	1404	1.14	285	2.42	0.14	0.081	5.56
11	570	1404	1.14	285	2.35	0.13	0.076	5.51

* CEM—Portland cement, ** SP—Superplasticizer.

**Table 6:** Experimental design conditions for investigating water demand and splitting tensile strength.

Technological Factors		Variation Levels			Variation Interval
Natural Value	Coded Value	−1	0	+1	
Maximum aggregate particle size, mm (Dmax)	X_1_	0.5	1.5	2.5	1
Portland cement–sand ratio (CEM/Sand)	X_2_	0.33	0.415	0.5	0.085

**Table 7:** Experimental design matrix for investigating water demand and splitting tensile strength.

No.	Coded Values		Natural Values	
	X_1_	X_2_	Dmax, mm	CEM/Sand
1	1	1	2.5	0.5
2	1	−1	2.5	0.333
3	−1	1	0.5	0.5
4	−1	−1	0.5	0.333
5	1	0	2.5	0.416
6	−1	0	0.5	0.416
7	0	1	1.5	0.5
8	0	−1	1.5	0.333
9	0	0	1.5	0.416
10	0	0	1.5	0.416
11	0	0	1.5	0.416

**Table 8:** Mixture compositions and experimental results for investigating water demand and splitting tensile strength.

No.	Mixture Composition, kg/t			Water Content, *l*/t	W/C	Splitting Tensile Strength, MPa	Standard Deviation, s	Standard Error, $s_{\bar{x}}$	Coefficient of Variation, V, %
	CEM	Sand	SP						
1	333	666.3	0.67	140	0.42	2.12	0.20	0.118	8.69
2	248	751.5	0.50	125	0.50	1.72	0.14	0.078	7.31
3	333	666.3	0.67	162	0.49	2.65	0.18	0.101	6.19
4	248	751.5	0.50	140	0.56	2.05	0.18	0.106	8.15
5	293	706.4	0.59	135	0.46	2.23	0.11	0.064	4.80
6	293	706.4	0.59	160	0.55	2.44	0.14	0.082	5.56
7	333	666.3	0.67	150	0.45	2.81	0.26	0.150	8.47
8	248	751.5	0.50	137	0.55	2.25	0.21	0.123	8.58
9	293	706.4	0.59	144	0.49	2.60	0.17	0.096	6.07
10	293	706.4	0.59	146	0.50	2.55	0.14	0.078	5.05
11	293	706.4	0.59	145	0.49	2.58	0.17	0.100	6.45

## Data Availability

The original contributions presented in this study are included in the article. Further inquiries can be directed to the corresponding author.
